# Sustainability Awareness in Manufacturing: A Review of IoT Audio Sensor Applications in the Industry 5.0 Era

**DOI:** 10.3390/s25103041

**Published:** 2025-05-12

**Authors:** Stefania Ferrisi

**Affiliations:** Department of Mechanical, Energetic and Management Engineering, University of Calabria, Ponte P. Bucci, 87036 Rende, CS, Italy; stefania.ferrisi@unical.it

**Keywords:** machining, predictive maintenance, audio signals, acoustic emissions, bibliometric analysis

## Abstract

The integration of Internet of Things audio sensors with Artificial Intelligence techniques is revolutionizing predictive maintenance systems in machining operations, playing a pivotal role in advancing the sustainability goals of Industry 5.0. The synergy between these technologies enhances operational efficiency, reduces downtime, and minimizes waste, aligning with energy conservation and resource optimization goals. The use of audio sensors provides a cost-effective, non-intrusive solution for machining operations. In this work, a bibliometric analysis of the progress achieved in this field is performed, identifying which challenges have been extensively addressed and which remain unexplored. By assessing the existing research, this study aims to highlight gaps that necessitate further investigation, guiding future research efforts toward the most critical and promising directions for enhancing predictive maintenance in machining processes. Through a comprehensive analysis of publication trends, collaboration networks, and research gaps, this study intends to provide valuable insights for academia and industry stakeholders, to motivate their efforts in this field. Understanding these trends is essential for fostering innovation and ensuring that the development of predictive models continues to evolve to maximize both production efficiency and sustainability.

## 1. Introduction

Sustainability has emerged as an important aspect of industrial processes. At a global scale, there is growing pressure to reduce carbon emissions from production; a challenge which is addressed by the simultaneous pursuit of enhanced energy efficiency, reduced waste, reduced costs, and increased process safety. With the advent of Industry 4.0, the integration of Internet of Things (IoT) sensors and Artificial Intelligence (AI) techniques had already begun to be employed to enhance manufacturing processes. This combination facilitated the monitoring, prediction, and identification of anomalies, contributing to improvements in production efficiency and, consequently, sustainability. Sustainable Manufacturing can be defined as the integration of processes and systems capable of producing high quality products and services using less and more sustainable resources (energy and materials), being safer for employees, customers, and surrounding communities, and being able to mitigate environmental and social impacts throughout the whole life cycle [1]. The implementation of sustainable manufacturing practices is based on the recognition that businesses and industries must operate in harmony with the environment. This is achieved through the integration of sustainable manufacturing as a strategic imperative [2]. In the age of Industry 4.0, with the use of modern smart technologies, manufacturing sustainability has evolved into a societal philosophy, and a dominant attitude for framing social, ecological, and ethical issues [3], termed Sustainability 4.0. More recently, in the era of climate change and increasing global environmental challenges, the latest industrial evolution, named as Industry 5.0, marks a turning point, from an industrial approach that focuses on efficiency and automation, to a paradigm that prioritizes collaboration between humans and machines, innovation, and harmonization with the environment [4]. This new paradigm has becoming a core enabler for achieving sustainable development and green growth in manufacturing.

García-Muina et al. [5] introduced a sustainability-focused business model canvas for an Italian ceramic tile company. Based on data collected through sensors and meters invested in by the company, their new business model enables the adoption of sustainable practices, including the reuse of consumed water, within the company’s value creation strategy. Furthermore, it suggests the implementation of other sustainability practices, in accordance with the sustainable transition of all enterprises.

The use of IoT-wearable devices allows enterprise to increase their work safety level, performing four different functions [6]: monitoring workers’ vital and environmental parameters [7], enabling communication [8], supporting training of workers using virtual and augmented reality [9], and tracking workers’ positions to prevent accidents [10,11]. In addition, Predictive Maintenance (PM) represents a transformative application of IoT in industrial settings. IoT devices and sensors can help predict when equipment is likely to fail, enabling proactive maintenance to prevent accidents caused by faulty machinery [12]. This approach minimizes downtime, reduces maintenance costs, and enhances worker safety by identifying and addressing issues before they escalate. The integration of PM with IoT has dual perspectives: it aligns with the goals of sustainability, and highlights the importance of advancing safety and operational efficiency across industries.

The implementation of a data-driven PM solution has attracted significant interest in modern manufacturing. Mallioris et al. [13] provided a systematic review of PM applications across industries, including machinery, transport, energy, chemicals, pharmaceuticals, and electronics. A decision support map was provided, with the aim of guiding researchers and technicians in the construction of PM applications for each respective industry, by suggesting common input features, methods/algorithms, and software tools. Joshi and Sharma [14] conducted an examination of the applications of Machine Learning (ML) algorithms in the context of low-carbon smart manufacturing, highlighting their role in integrating sustainable practices. The implementation of these advanced technologies, such as ML and Deep Learning (DL) algorithms, requires the collection of real-time data from the manufacturing processes. The integration of IoT devices and sensors facilitates the establishment of a connection between machines and algorithms, enabling the collection of real-time data from the four distinct product life cycle stages, namely supply, production, use, and end of life [15]. This, in turn, enables enhanced decision-making processes, leading to improvements in operational efficiency and the sustainability of manufacturing processes. Data collected during production can facilitate predictive maintenance by continuously monitoring the health of machines and equipment [12] and the quality of the machined parts [16].

**Figure 1:**
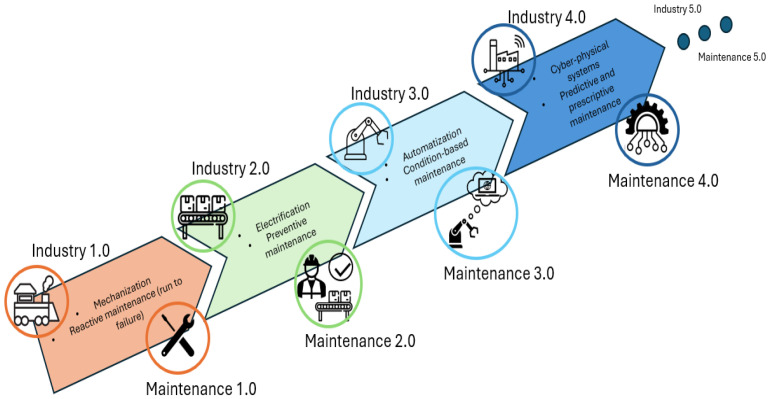
Development of maintenance paradigms.

In Industry 4.0, sustainability has reshaped smart manufacturing, giving rise to Maintenance 4.0 [17] (also called Smart Maintenance), evolving from the reactive Maintenance 1.0 to the predictive and perceptive Maintenance 4.0 [18]. Maintenance 1.0 relied on operators fixing simple machinery faults as they occurred. Industry 2.0 introduced more complex machinery, necessitating dedicated maintenance departments for preventive care and inspections. The advent of automation has precipitated a concomitant shift in maintenance strategies, with the implementation of condition-based maintenance (or Maintenance 3.0), becoming the dominant paradigm. In this novel paradigm, maintenance models are constructed on the basis of collected and analyzed data, with the subsequent generation of recommended maintenance actions, predicated based on the specifics of the situation. Finally, with the advent of Industry 4.0, the use of IoT devices and sensors and the prominence of sustainability concepts evolved into the Maintenance 4.0 paradigm, in which maintenance becomes an enabler for the smart factory [19], by predicting future failures and prescribing efficient preventive actions. As Industry 5.0 emerges, maintenance must align with its core principles, progressing toward Maintenance 5.0. The progression of maintenance paradigms as the industry advances is illustrated in Figure 1.

**Figure 2:**
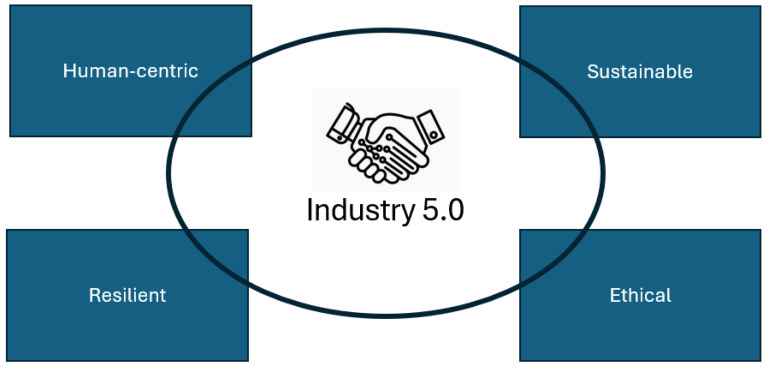
Core values of Industry 5.0.

The transition to the new Industry 5.0 paradigm has defined modern organizations, demonstrating a shift and increase in focus, towards ethical engineering principles [20]. It has introduced new views of sustainability, such as social, which focus on the well-being of operators. Therefore, the use of smart machines equipped with sensors is becoming essential for the construction of automated tools capable of reducing the pressure on operators in managing emergency situations such as extraordinary maintenance. All maintenance efforts in this paradigm are guided by the four values of Industry 5.0, as illustrated in Figure 2.

This literature analysis has highlighted the maintenance-related impacts on the three aspects of sustainability: environmental, economic, and social [17,18]. Table 1 provides a summarization of these impacts.

All these positive impacts can be achieved by combining IoT devices and/or sensors and ML or DL techniques to implement a Maintenance 5.0 strategy. Furthermore, the costs associated with IoT monitoring systems can be onerous for companies, especially small- and medium-sized enterprises [21]. For these companies, the initial investment for IoT infrastructure can be prohibitively expensive [22].

**Figure 3:**
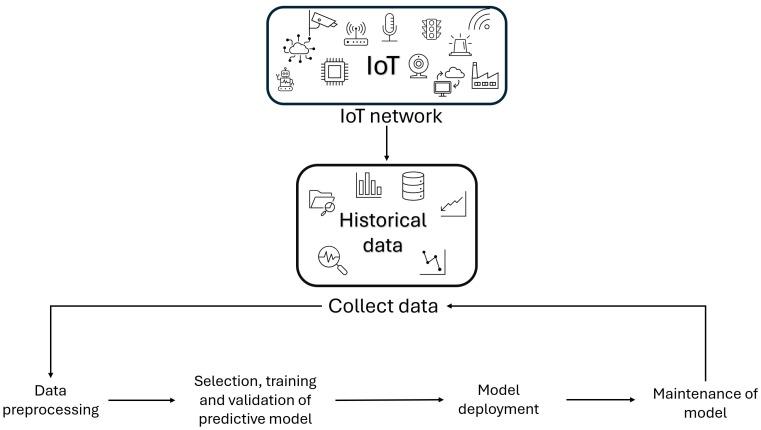
Framework for the development of an IoT and AI-based PM model.

The implementation of an IoT network that has been customized for industrial processes is a prerequisite for the subsequent application of ML or DL techniques to sensor data to formulate a predictive maintenance strategy. This process is characterized by four distinct stages [23], as illustrated in Figure 3. The first stage consists of the selection of historical data, collected through sensors. Subsequently, the selected data must be pre-processed, in order to transform them into an appropriate form for the next stage, in which a predictive model is constructed to recognize future failures. Finally, there is the maintenance stage of the model, in order to maintain the performance of the model over the long term, and the acquisition of new data.

**Figure 4:**
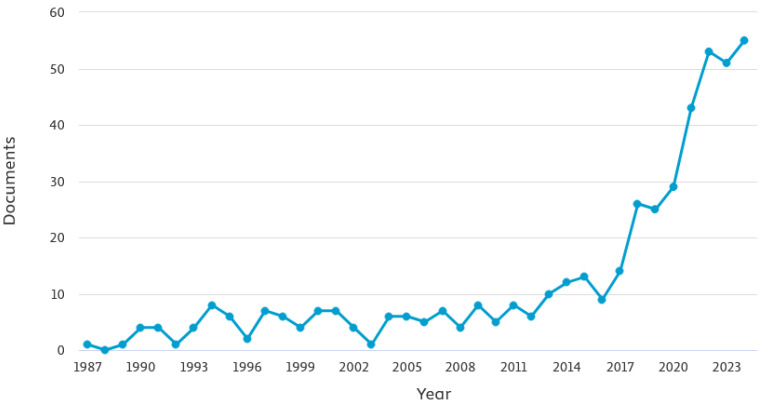
Number of documents published per year in Scopus (1986–2024) based on the following query: (TITLE-ABS-KEY (audio OR sound OR “acoustic emissions” OR microphone) AND (“machine learning” OR “deep learning” OR “artificial intelligence” OR “neural network”) AND (machining)).

In the domain of machining operations, the most frequently used sensors are the following: accelerometers, which are utilized for the acquisition of vibrations; dynamometers, which are employed for the measurement of cutting force; and microphones, which are used for the purpose of recording audio [24]. To construct PM systems at minimal financial cost, microphones may be the most cost-effective sensor to employ. Given this advantage, combined with the demonstrated prediction performance of a system based on audio signals for predicting future failures in an industrial setting, interest in this field has grown rapidly in recent years, as shown Figure 4.

This growing interest has led to the publication of numerous works exploring vastly different directions, making it challenging to identify or develop an adequate solution for practical applications. Different maintenance-related standards have been identified from organizations such as the International Standards Organization (ISO) and American National Standards Institute (ANSI). In the literature, a wide range of factors, such as sampling rate, microphone positioning, and cutting parameters, have been considered, leading to varied results and approaches.

This review article aims to provide a comprehensive overview of the research conducted in the field of predictive maintenance for machining operations, focusing on approaches that combine AI techniques with data acquired from sensors, particularly through the use of microphones for capturing audio signals. The main objective is to ensure that researchers and enterprises gain a clear understanding of how audio signals can be leveraged for real-time monitoring, control, and optimization of industrial processes. Additionally, this review highlights the potential of predictive maintenance systems based on AI and audio signals, examining key applications such as anomaly detection for predicting anomalies during production, fault detection for forecasting machine failures, and tool condition monitoring (TCM) of the tools used in the process. Furthermore, the review will assess which of the different machining processes scientific research is focusing. By clarifying the various applications and settings analyzed in existing research, this article aims to provide a clearer picture of the current state of the field and offer guidance on the direction of future research in predictive maintenance.

The rest of this article is organized as follows: Section 2 provides a detailed explanation of the methodology adopted for the selection of the papers analyzed in this review work. The section is divided into four subsections, corresponding to the four phases of the PRISMA methodology. A further subsection relates to the formulation of additional research questions. Section 3 presents the results of the analysis, while Section 4 provides a discussion of the findings of this review work. Finally, Section 5 provide the conclusions of the study.

## 2. Research Methodology

The present work is a literature review of the most recent research works focused on the definition and construction of PM systems based on the combination of audio signals and AI techniques. To conduct a literature review, several methodologies can be used. The most common methodology used is Preferred Reporting Items for Systematic Reviews and Meta-Analyses (PRISMA) [25], which helps in identifying an evidence-based minimum set of bibliographic records to be reported in systematic review studies and meta-analyses. It was designed to help reviewers in transparently reporting why a review was carried out.

It considers four main phases:

1. Identification: this is the most important step in a literature review, in which a clear definition of the questions that the reviewer wants to answer must be defined. After this, defined queries are used to find papers in databases such as Scopus and Web Of Science;
2. Screening: in this phase, certain database papers are removed, based on inclusion or exclusion criteria;
3. Eligibility: another screening is carried out to decide whether a paper is relevant or not. Duplicates and papers with missing data or information are removed;
4. Inclusion of studies: manual inclusion of missing publications not found using the search query is carried out.

**Figure 5:**
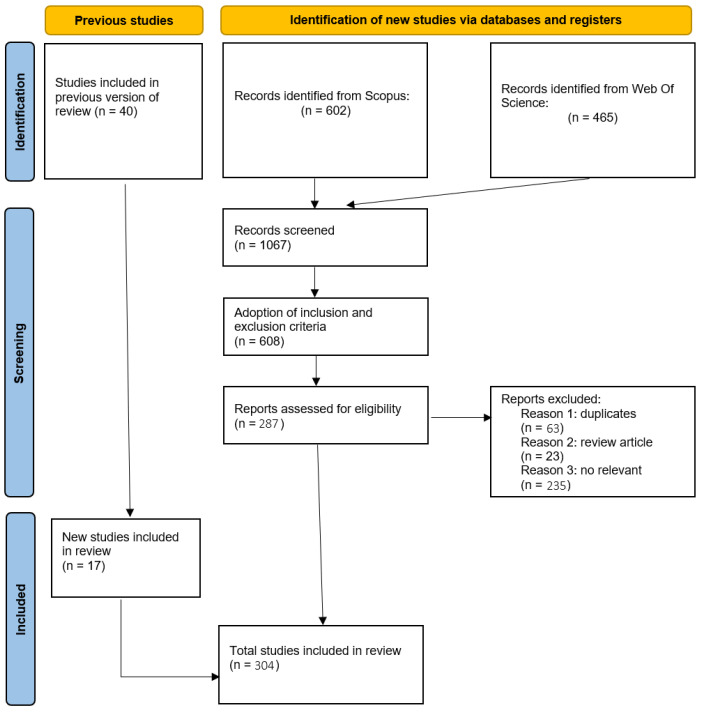
Flow diagram for searching and selecting papers, adapted to the PRISMA methodology [25].

As delineated in the following subsections, the execution of these phases resulted in the selection of 304 papers for the analysis, as illustrated in Figure 5.

### 2.1. Identification Phase

**Figure 6:**
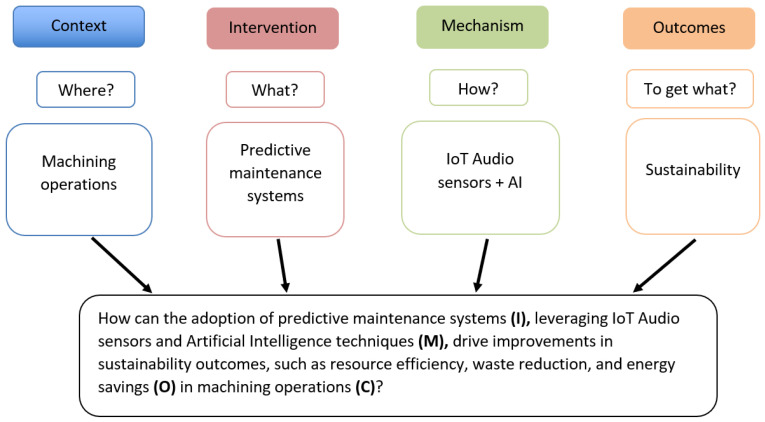
Formulated research question based on the CIMO-logic.

In this study, the CIMO (Context, Intervention, Mechanism, and Outcome) methodology [26] was used to formulate the research question. The formulated research question, constructed as shown in Figure 6, was as follows:

How can the adoption of predictive maintenance systems, leveraging IoT audio sensors and Artificial Intelligence techniques, drive improvements in sustainability outcomes, such as resource efficiency, waste reduction, and energy savings in machining operations?

Once the research question has been clearly defined, a list of keywords was defined for each element of the CIMO framework, as shown in Table 2.

From the research question and the list of keywords identified, the following query was used from the Scopus and Web Of Science databases, by searching in the Title, Abstract, and Keywords:
(machining OR milling OR turning OR drilling OR threading OR sawing OR grinding OR “tool wear” OR “cutting tool” OR CNC OR “industrial machine*”)AND(“predictive maintenance” OR “Condition based maintenance” OR Monitor* OR ((fault OR failure OR anomaly) AND (prediction OR detection)))AND(“acoustic emissions” OR audio OR sound OR microphone)AND(machine*learning OR ML OR “artificial intelligence” OR “deep learning” OR “neural network*” OR ai OR NN OR “data driven” OR LSTM OR prediction OR CNN OR RNN)AND(sustainab* OR green OR “circular economy” OR efficien* OR waste OR decarbonization OR “triple bottom line” OR TBL OR carbon OR emissions)

A total of 602 and 465 papers were identified in the Scopus and Web of Science databases, respectively. The period over which the publications span is from 1984 to 2024.

### 2.2. Screening Phase

In this phase, some papers were removed, based on inclusion and exclusion criteria listed in Table 3. These criteria were chosen with respect to the research question, with the aim of excluding papers that provided no information or did not emphasize the latest solution approaches for the field under investigation. In detail, to include all the latest research advancements in the use of IoT audio sensors for predictive maintenance in manufacturing, the past decade was chosen as the analyzed period. Subsequently, a preliminary list of inclusion fields was selected from Scopus, with the objective of determining those that would be the most relevant to the thematic focus of the study. Subject areas pertaining to engineering, informatics, and material science were incorporated to encompass all aspects of the utilization of IoT audio sensors for predictive maintenance (e.g., smart manufacturing, machining, and AI applications). Furthermore, fields such as decision science and operational research were incorporated, to encompass subjects related to optimization and resource management, in accordance with the principles of Industry 5.0. A selection of subject categories in Web of Science was consequently made to cover the same aspects highlighted in Scopus. The aims and scope of the journals contained in these categories were examined in detail, in order to ensure that the most appropriate selection was made based on the aspects that needed to be covered. The intention was to obtain a comprehensive overview of the topic under analysis in this paper.

A total of 278 papers from Scopus and 330 papers from Web of Science were obtained. The number of review articles obtained from each database was 8 and 15, respectively, and Web of Science additionally provided two data papers.

### 2.3. Eligibility Phase

In this phase, another screening was performed, deciding if a paper was relevant or not. Initially, duplicates from both databases were removed. A total of 545 unique papers (63 duplicates) were obtained from the merging of the two databases. Subsequently, the review articles were isolated and analyzed separately from the other types of articles, on which bibliometric analyses were conducted. This process resulted in a total of 523 articles being selected for further analysis. A final screening phase was conducted to ensure the quality of the papers. This phase involved a review of the titles and abstracts of the papers to ascertain their relevance, as well as whether they were related to the field analyzed, i.e., work on predictive maintenance in machining operations. To minimize bias, this final screening was repeated twice at an interval of two weeks, allowing for a critical re-evaluation of inclusion and exclusion decisions. At end of this procedure, a total of 287 papers were selected.

### 2.4. Inclusion of Studies

In the last phase of inclusion of studies, the missing publications not identified by the search query were manually included. In this review, other relevant research works on the field addressed were included. The identification of omitted papers was based on a previous review by the author of this work [27]. In particular, 17 other papers were incorporated, thereby yielding a total of 304 papers.

### 2.5. Other Research Questions

Following the PRISMA methodology, articles addressing the main objective of Industry 5.0 of improving the sustainability of manufacturing processes by applying predictive maintenance strategies were selected. Subsequently, additional research questions were formulated to analyze the trends and advancements in this field.

These questions aimed to provide deeper insights into the directions undertaken by researchers and to identify potential gaps for future exploration. The motivation for this analysis stemmed from the need to understand how predictive maintenance, supported by IoT and AI technologies, contributes to achieving sustainable outcomes in manufacturing. By answering these questions, the review sought to clarify the progress made and highlight opportunities for innovation in this critical domain.

The questions with their significance are given in Table 4. They are intended to identify actionable insights for advancing the field, providing a clear understanding of the state of the art and the remaining challenges. These insights are crucial for aligning research with the broader goals of Industry 5.0, which emphasizes the integration of smart technologies to achieve sustainable, efficient, and resilient manufacturing systems.

## 3. Results

This section provides a concise and precise description of the findings of our work: (i) descriptive results about the number of works examined and the scientific production evolution are provided; (ii) several bibliometric analyses were carried out; (iii) a clustering analysis of the main themes addressed in the analyzed field was conducted; (iv) publicly available datasets used in the analyzed papers were identified.

### 3.1. Descriptive Results

**Figure 7:**
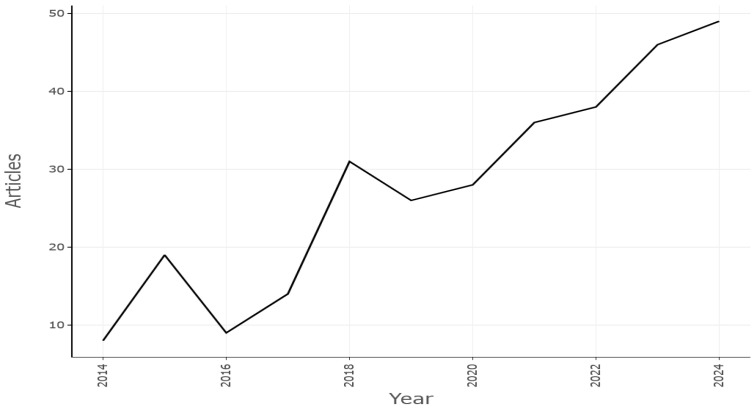
Scientific production from 2014 to 2024.

The analysis involved 304 documents published from 2014 to 2024, with an annual growth rate of 19.87% and a distribution as shown in Figure 7. The number of authors engaged was 984 and an average citation of 18.91 per document was obtained. The average number of citations, normalized per year, was computed as follows:

(1) NACY=Totalcitationsforallpaperspublishedintheyear/NxCY

where:

- *NACY* = Normalized Average Citation per Year,
- *N* = number of papers published in a given year,
- *CY* = number of years since the papers were published, up to the present year.

**Figure 8:**
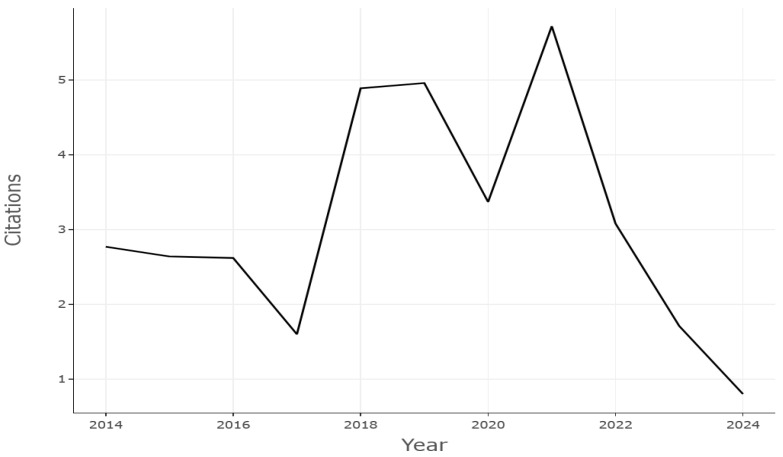
Average citation of papers per year.

Papers from 2018 to 2022 were most cited, with the highest values of NACY, as shown in Figure 8.

**Figure 9:**
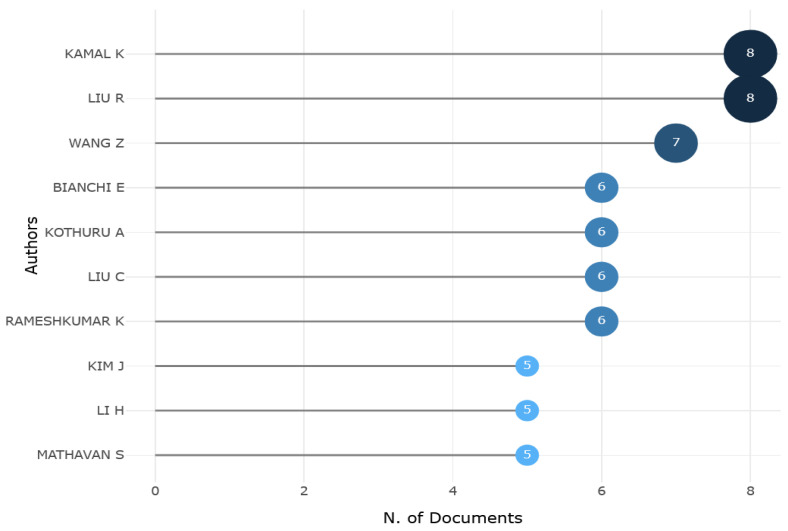
High-contributing authors.

**Figure 10:**
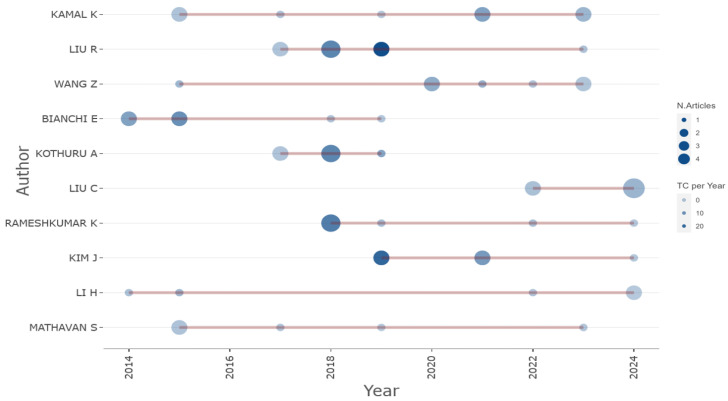
High-contributing author production.

**Figure 11:**
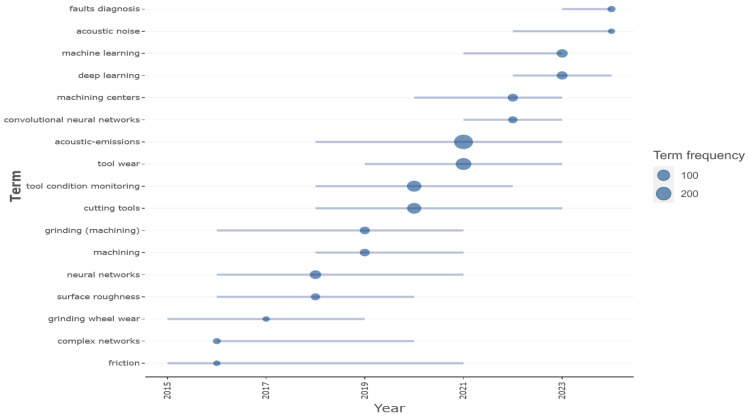
Trend topics over years.

High-contributing authors are depicted in Figure 9. An analysis of production data over time reveals that the majority of production occurred during the period from 2018 onward (See Figure 10), in accordance with peaks depicted in Figure 8. These authors are strongly interested in the field of tool condition monitoring and the influence of tool wear on production efficiency. Indeed, as Figure 11 shows, a transition towards tool condition monitoring topics emerged during the period between 2018 and 2022.

**Figure 12:**
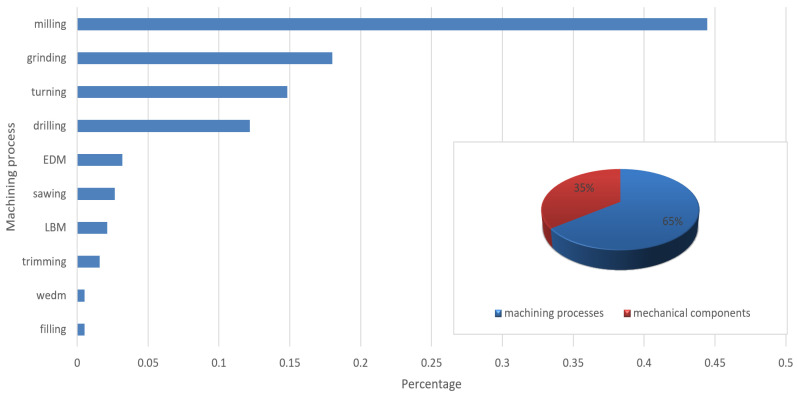
Machining processes analyzed.

The findings demonstrated a high level of scientific interest in the utilization of audio signals in domains such as anomaly detection and TCM for machining operations. The construction of predictive models aimed to achieve objectives including enhanced process efficiency, reduced waste, minimized machine downtime, and, consequently, enhanced sustainability. As shown in Figure 12, 65% of the studies addressed various machining processes, whereas 35% focused specifically on individual components of industrial machinery, such as pumps and gearboxes, with the milling process being the most analyzed, followed by the grinding process.

### 3.2. Bibliometric Analysis

Bibliometric analysis is a quantitative method that is utilized to analyze large volumes of scientific literature and to identify impactful studies, authors, journals, organizations, and research trends. The goal of a bibliometric analysis is to summarize large quantities of bibliometric data to present the state of the intellectual structure and emerging trends in a research topic or field [28]. It is widely used when a dataset is too large for a manual review. Different techniques can be applied to conduct a bibliometric analysis. Among them, Bradford and Lotka’s laws, country collaborations, Wordclouds, and citation analysis were used in this review work to identify the top journal, articles, and countries engaged in the scientific production in this field. To visualize the results of the bibliometric analysis, the command-based software of the Bibliometrix package in R [29] was used.

#### 3.2.1. Bradford’s Law

Bradford’s law describes the distribution of citations for a given subject or field. Its application enables the identification of the most highly cited journals within a given field or subject. It provides a division into three zones of journals, from a “core zone”, which contains the most relevant and widely cited articles, to Zone 3. By applying this technique separately for the publication types of journals and conference proceedings, a list of those falling within the “core zone” was created, as shown in Table 5.

#### 3.2.2. Lotka’s Law

Lotka’s law is another type of distribution that describes the productivity of authors in a given field. In accordance with this law, a greater proportion of authors contribute with a limited number of articles, typically one or two, while only a small proportion of authors contribute with a significant amount of publications. This pattern has been observed to be consistent across various academic disciplines, following an inverse square law. This means that, for a given X authors who have written one paper, the proportion of those writing two papers is only X/4, the proportion of those writing three papers is X/9, and so on.

**Figure 13:**
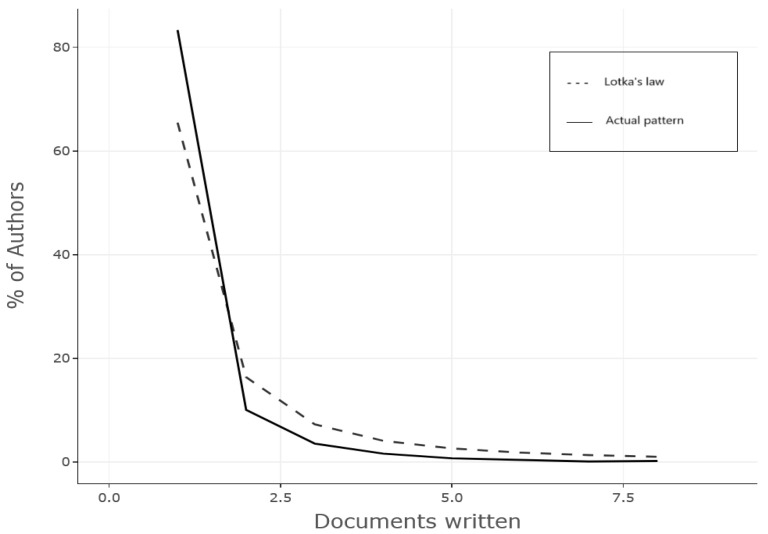
Author productivity by Lotka’s law.

A comparison of Lotka’s law and the actual distribution of author productivity, as illustrated in Figure 13, revealed that the field under investigation in this study predominantly adhered to this distribution, with 80% of authors having published only a single article, while only a negligible proportion had published more than five articles.

#### 3.2.3. Keywords Analysis

**Figure 14:**
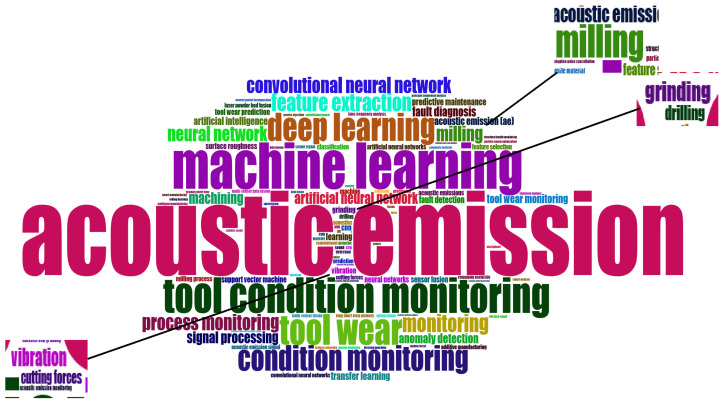
WordCloud.

From the identification of the most relevant terms, as shown in Figure 14, it was immediately observed that the tool wear condition monitoring is the problem most frequently addressed in the extant literature. Moreover, of all the machining processes, milling is the most discussed and analyzed, followed by grinding and drilling. Regarding the ML/DL components, neural networks emerged as the most widely used technique, especially the convolutional type, suggesting a significant use of transformation of audio data in images. This is followed by the support vector machine model. As is evident from the WordCloud, there is a necessity to process audio signals in order to transform them into a format suitable for the implementation of ML or DL techniques. In addition, force and vibration signals emerged as other types of signals used in addition to the more common acoustic emissions.

**Figure 15:**
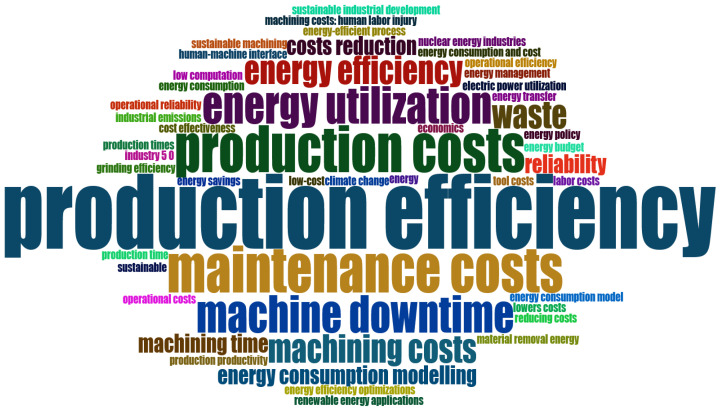
Sustainable WordCloud.

A more in-depth analysis of the most relevant terms was conducted, focusing exclusively on keywords associated with the sustainability goals across all three dimensions of economic, environmental, and social sustainability. A new WordCloud, as illustrated in Figure 15, was obtained. The analysis revealed a predominant emphasis on the economic and environmental dimensions, a focus likely driven by the higher returns companies expect from such investments. In contrast, relatively few studies addressed social aspects, which can be attributed to the recent emergence of the Industry 5.0 paradigm. This disparity highlights a critical gap: despite growing global calls for greater attention to sustainability issues, research on the social dimension remains limited. Therefore, further efforts are needed to advance its integration into both industrial practice and academic efforts.

#### 3.2.4. Countries’ Scientific Production

**Figure 16:**
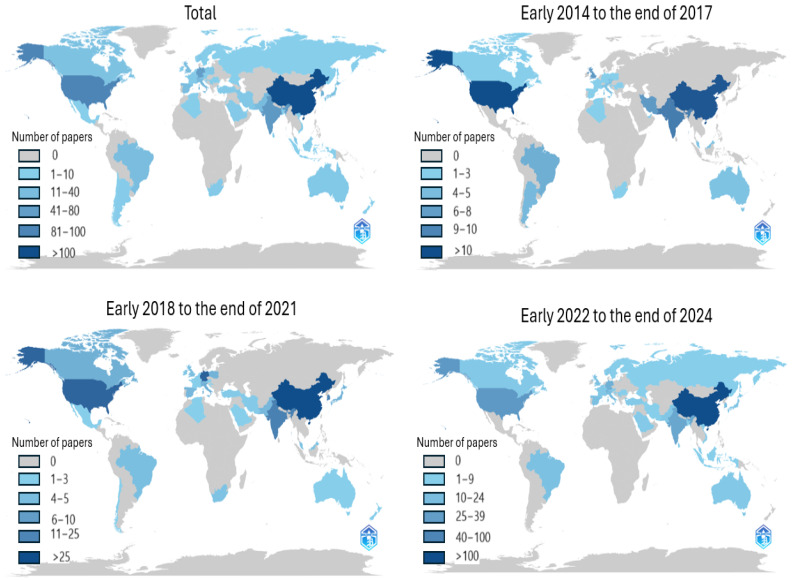
Countries’ scientific production over time.

**Figure 17:**
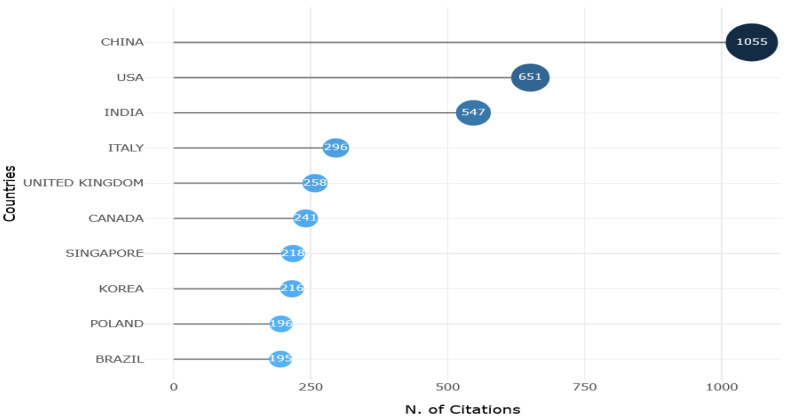
Most cited countries.

By quantifying the number of publications across different countries and time periods, the dominant players in the scientific field analyzed and the emergence of new contributors were determined, as shown in Figure 16. The analysis revealed that countries such as the United States, China, and Germany have consistently led in terms of research output. A notable trend is the rapid rise in research output from China. A similar trend was observed in some countries of South Asia, South America, and some parts of Africa, which indicates an increase in investments in research infrastructure and international collaborations. A comparison of the three periods showed a sharp increase in productivity. The years 2018–2021 marked a surge in publications from emerging economies, suggesting a democratization of scientific knowledge production. Furthermore, an examination of the number of citations received by countries, as illustrated in Figure 17, revealed that significant contributions to scientific knowledge were also made by other countries, including India, Italy, and United Kingdom.

**Figure 18:**
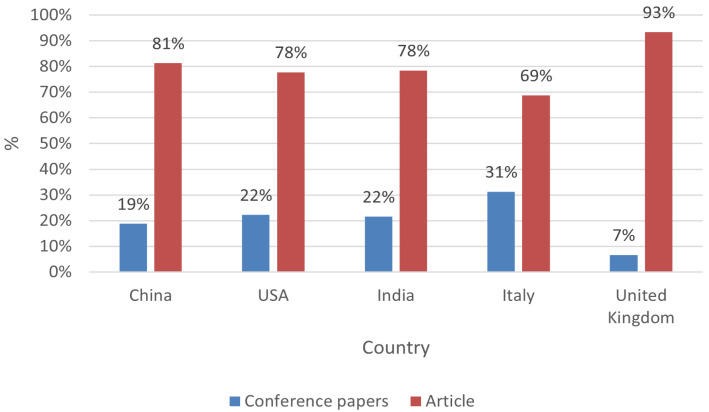
Types of papers published for the five most cited countries.

A more detailed analysis of the five most cited countries showed that journal articles represented the majority of publications for all themes, with the same distribution for the top three countries (i.e., China, USA, and India), while Italy had a slightly higher rate of conference papers, and the United Kingdom had the most unbalanced distribution, as shown in Figure 18.

**Figure 19:**
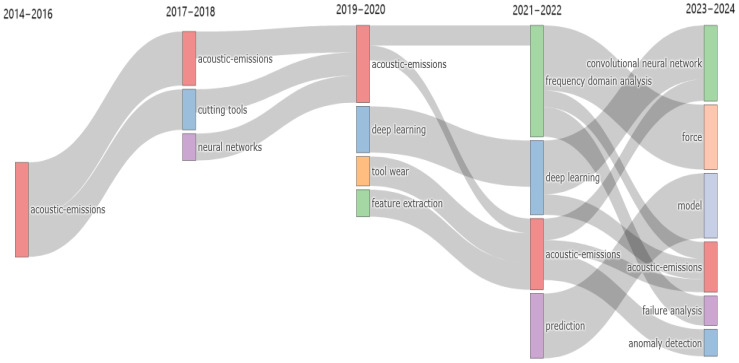
Thematic evolution for China.

**Figure 20:**
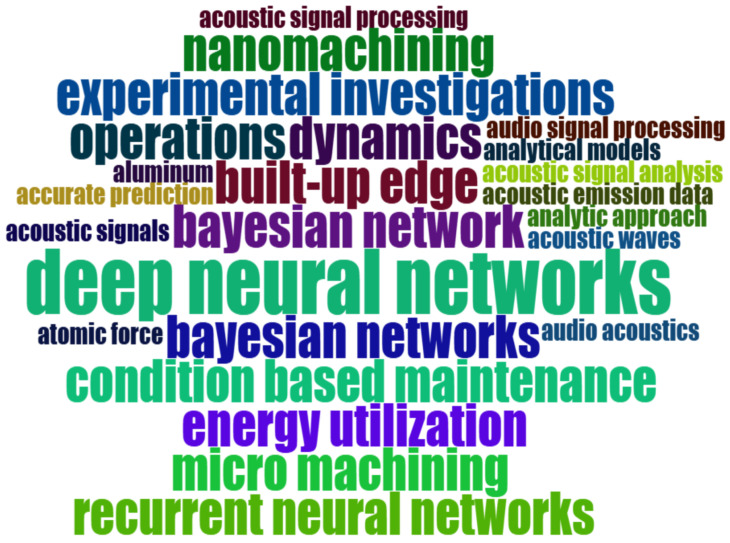
WordCloud for USA.

By focusing on the thematic trend of each country, an evolution emerged for China, as shown in Figure 19. As acoustic emission acquisition remained a key topic throughout the decade, studies up to 2021 mainly focused on tool condition monitoring and the prediction of tool wear, while later years showed a change in the trend, with the introduction of failure prediction as a problem to be solved. Conversely, the USA did not show a change in the thematic trend, but studies mainly focused on the use of Bayesian optimization to identify the optimal configuration of neural network architecture, as shown in the WordCloud in Figure 20.

**Figure 21:**
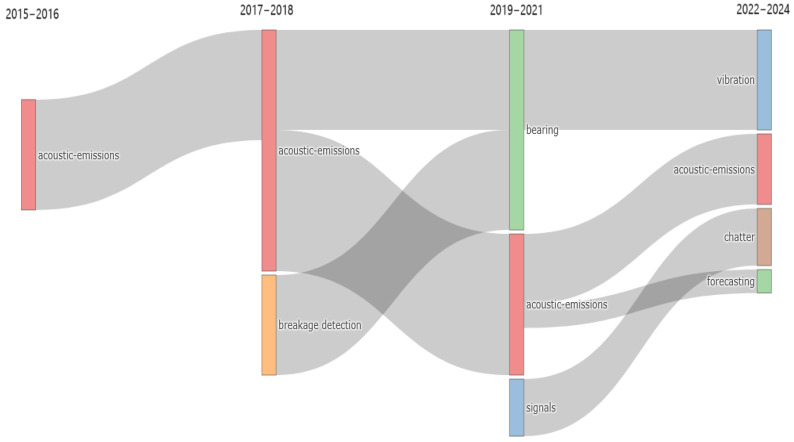
Thematic evolution for India.

Figure 21 shows the thematic specialization over time for India. It shows that acoustic emission acquisition, and consequently signal analysis, has been mainly used for breakage detection of mechanical components, especially for bearings, in contrast to the other countries, which have mainly focused on tool condition monitoring. In addition, in recent years, research topics such as vibration and chatter indicate an increased focus on the detection of unstable phenomena during machining processes.

**Figure 22:**
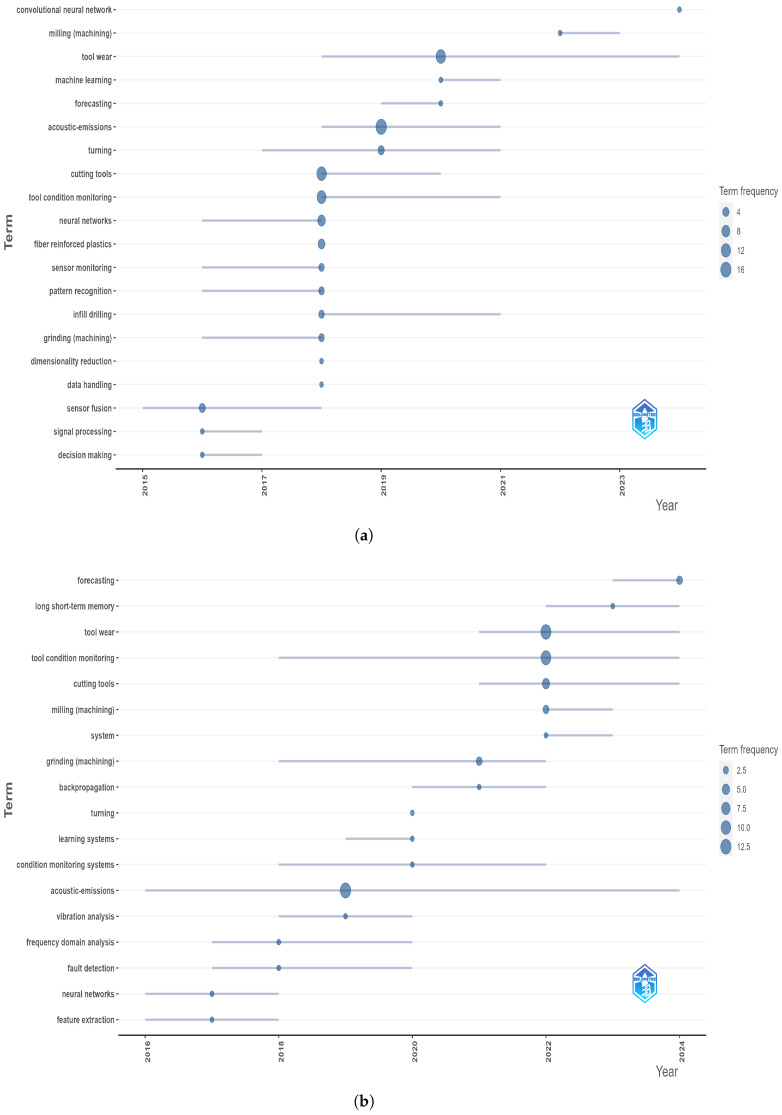
Trend topics for (**a**) Italy and (**b**) United Kingdom.

By analyzing the trend topic diagrams for both Italy and the United Kingdom in Figure 22, it can be seen that in both countries there has been a clear evolution in the research themes over time. In particular, the evolution mainly concerns the types of manufacturing processes investigated, with milling processes only receiving attention in recent years. Overall, both countries showed a positive trend towards greater specialization, but with a noticeable difference in the speed of thematic development. In the case of the United Kingdom, the evolution was more rapid and diversified, with research themes expanding and specializing within shorter time intervals. This suggests a dynamic research environment, where emerging technologies are quickly incorporated into scientific investigations. Conversely, the thematic evolution in Italy appeared to be slower and more gradual. While new topics were introduced over time, the focus tended to remain on established topics for longer before moving on to new areas of research. This slower transition may reflect a more conservative research trajectory, possibly emphasizing the consolidation and refinement of existing methodologies before embracing new directions.

**Figure 23:**
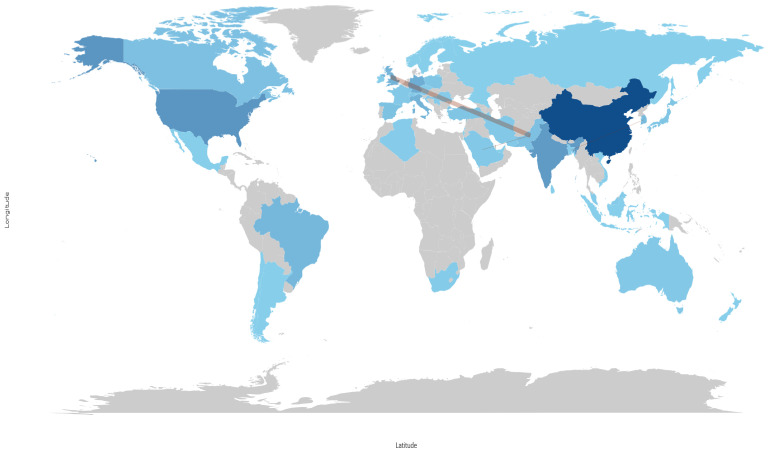
Countries’ collaboration map.

Despite the high level of interest in the field worldwide, only a very small proportion (around 10%) of publications were produced by a collaboration, as shown in Figure 23.

#### 3.2.5. Most Cited Articles

As shown in Figure 8, the most cited articles are concentrated between 2018 and 2021. This suggests that research published during this period has garnered significant attention within the academic community, potentially due to its relevance, novelty, or alignment with emerging trends in the field. Table 6 lists the top 10 articles for total citations per year.

### 3.3. Thematic Map

A Thematic Map is a visual tool to map topics addressed in the analyzed field, by identifying clusters of keywords. These clusters are considered themes and are classified into four categories:

- Niche themes: the themes in this cluster are well developed but not particularly important for the research field analyzed;
- Motor themes: the keywords in this cluster identify topics important for the field under investigation;
- Emerging or declining themes: these themes are weakly used and are therefore identifiable as emerging if recently used or in decline;
- Basic themes: the themes in this cluster are important and concern general topics for the field under investigation.

**Figure 24:**
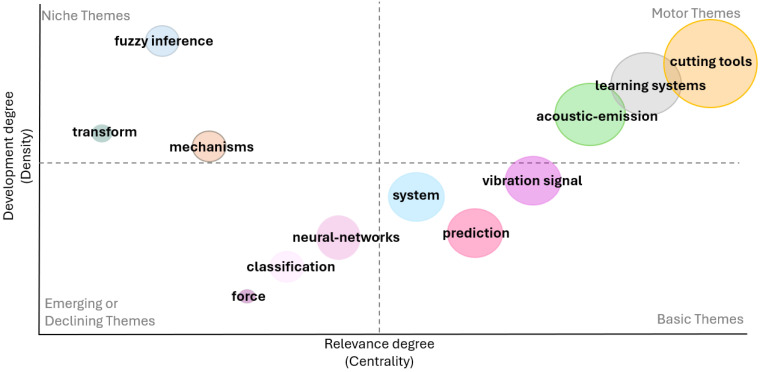
Thematic map.

Based on the analysis of the selected set of papers, the Thematic Map shown in the Figure 24 was generated.

**Figure 25:**
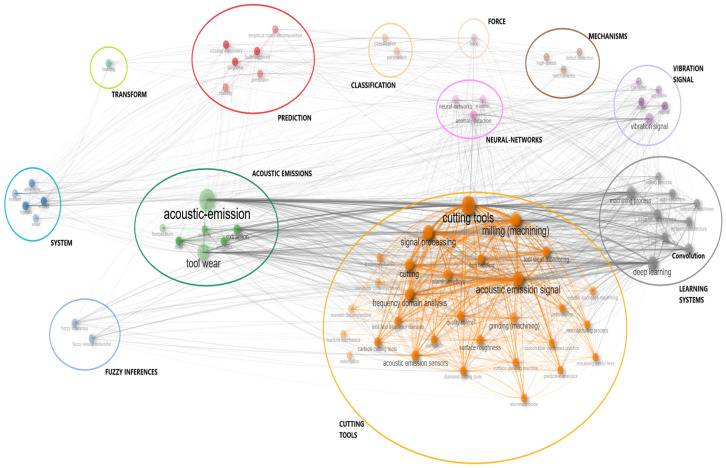
Cluster keywords.

The analysis yielded twelve clusters of keywords, the list of which is provided in Table 7, together with their respective meanings, as derived from the keywords contained within them (see Figure 25). The themes that were identified as either emerging or declining were characterized through an analysis of the associated keywords in papers during the specified period. For instance, the force theme, which pertains to the utilization of sensors for the capturing of force signals during machining processes, was determined to be a declining theme. This decline was characterized by the replacement of force sensors with alternative sensors capable of acquiring signals such as vibrations or acoustic emissions, which are considered more sensitive to changes in manufacturing processes than force signals.

A strong connection between these themes is highlighted in Figure 25, demonstrating the necessity of considering most of these themes in research activities. For instance, the analysis revealed the prevalence of themes such as cutting tools, acoustic emission, and learning systems in the majority of the articles examined. This observation suggests that these themes represent commonly used techniques that are necessarily employed together to produce scientific outcomes, making their integration an expected and essential aspect of research in the field.

Entering into these motor themes, a more in-depth analysis of the used techniques can be made. The most consistent theme was that related to the cutting tools monitoring problem. Machining processes depend on cutting tools to precisely remove material from a workpiece, thereby ensuring the final product’s quality. Consequently, the monitoring of cutting tools has emerged as a pivotal area of research. By detecting tool wear, failures, and optimizing tool usage, manufacturers can enhance process efficiency, minimize waste, and improve both productivity and sustainability. The analysis of keywords within the specified theme revealed the prevalence of acoustic-emission-related terminology, underscoring the extensive utilization of these signals in constructing predictive models for cutting tool issues. This suggests that acoustic emissions are very sensitive to tool changes.

Finally, the third motor theme was related to learning systems, encompassing keywords such as industrial machines and audio signals. These elements are fundamental to the construction of predictive models, as well as the ML/DL component, related to the use of specific, efficient algorithms. In this field, the DL component and its algorithms emerged. DL is a subset of machine learning that is inspired by the human brain, defining artificial neural networks (ANNs) to automatically learn patterns from large datasets. Among the various types of ANNs described in the literature, convolutional neural networks were the most applied in this field, as suggested from the relevant keyword ‘convolution’ (See Figure 25).

### 3.4. Publicly Available Datasets

The scarcity of readily available datasets constitutes a significant challenge in the field of predictive maintenance for industrial applications, particularly those pertaining to the acquisition of audio signals. Industry tends to be reluctant to share data, due to concerns regarding privacy and competitive advantage. This hinders scientific research, which requires substantial amounts of data to develop and evaluate new predictive methods with optimal prediction performance, particularly in terms of generalizability. An analysis of the data collection section in the papers selected by this review work revealed that only 12% of these obtained their results by testing their proposed methodologies on public datasets. Table 8 provides a list of the public datasets discovered from this analysis.

Furthermore, some studies have made the datasets generated during their research publicly available following the publication of their articles [72,73,74,75]. This practice contributes to the advancement of the field by enabling reproducibility and fostering the development of more robust predictive maintenance techniques.

## 4. Discussion

In this section, answers to research questions are discussed.

The main goal of this work was to provide a comprehensive overview of research endeavors centered on the development of predictive maintenance systems for machining processes, with a view to enhancing sustainability in accordance with the paradigm of Industry 5.0. Given the significant interest in this field, the present review focused on an analysis of studies related to the use of audio signals or acoustic emissions for the monitoring of machining processes. The decision to focus on these sensors was driven by the premise that they offer a cost-effective alternative to commercially available sensors, thus aligning closely with the sustainability objectives of the Industry 5.0 paradigm.

The present work aims to address a gap in the extant literature by providing an effective overview of the state of the art regarding the use of these sensors to improve machining processes. The high scientific interest in this field is evidenced by the publication of numerous review works, which have analyzed the use of sensors in general in some works, and more specifically the acquisition of audio signals or acoustic emissions in others. However, these works have focused on a single specific machining problem.

Kasiviswanathan et al. [76] examined papers pertaining to the utilization of sensors in conjunction with ML methodologies for the development of a predictive model for the issue of TCM in CNC turning centers. The authors recognized the necessity of defining a non-disruptive solution for standard industrial CNC machines, to integrate sensors without compromising the CNC machine’s efficiency and safety. In addition, Pimenov et al. [77] examined papers related to the TCM problem, in this case, for the milling process, and the authors recognized the high installation costs connected to the use of sensors like dynamometers or accelerometers to monitor the process. Conversely, they highlighted the necessity of combining sensors and ML techniques for the development of predictive systems in the context of sustainable manufacturing. Gawde et al. [78] published a systematic literature review related to the development of predictive models for fault diagnosis of rotating machines, analyzing the use of several sensors. Wei et al. [79] conducted a comprehensive examination of how ambient noise, specifically in high-speed cutting scenarios, impacts processing efficiency and accuracy. The review encompassed a detailed analysis of noise identification, signal acquisition, and noise processing methodologies employed in high-speed machining contexts. The study also examined tool condition and chatter detection works. However, it did not explore the integration of IoT audio sensors with advanced ML/DL techniques, nor did it address sustainability aspects that are crucial for Industry 5.0.

Our review aims to fill this gap by offering a bibliometric analysis and highlighting the evolution of the state of the art, emphasizing the convergence of intelligent audio sensing, AI algorithms, and green manufacturing strategies. Table 9 presents the exhaustive literature review, which is organized according to area, keywords, and time frame.

Building upon these prior reviews, the present work seeks to provide a broader overview of the current state of the art regarding the use of cost-effective and easily implementable sensors, specifically microphones for audio signal acquisition. Unlike other sensor technologies, microphones offer a non-intrusive solution that does not require modifications to the structural components of industrial machines, making them an attractive option for widespread adoption in manufacturing environments. This characteristic aligns with the principles of sustainability within the Industry 5.0 paradigm, as it facilitates the integration of advanced monitoring systems without requiring significant economic or operational burdens on manufacturers. Nonetheless, as revealed by the analysis in Section 3.3, the acquisition of acoustic emissions instead of audio signals was more frequently used in the literature. In any event, the utilization of audio signals or acoustic emissions, in conjunction with ML or DL techniques, has been demonstrated to be a more efficacious approach for the development of predictive models, with the objective of enhancing the sustainability of numerous machining processes. A powerful tool was obtained from this combination for the understanding of the erosion of the material in EDM [84]. High prediction performance was obtained by testing a deep architecture constructed for music and sound detection on a machine diagnosis dataset [85]. An efficient balance between accuracy and computational resource usage was demonstrated by testing a simple neural network for tool wear prediction in a milling process [66]. The scientific literature has demonstrated that the adoption of predictive maintenance systems, leveraging IoT audio sensors and AI techniques, can lead to improvements in sustainability outcomes for machining operations. These outcomes are achieved by addressing various industrial problems, including tool condition monitoring, fault diagnosis, and surface quality.

**Figure 26:**
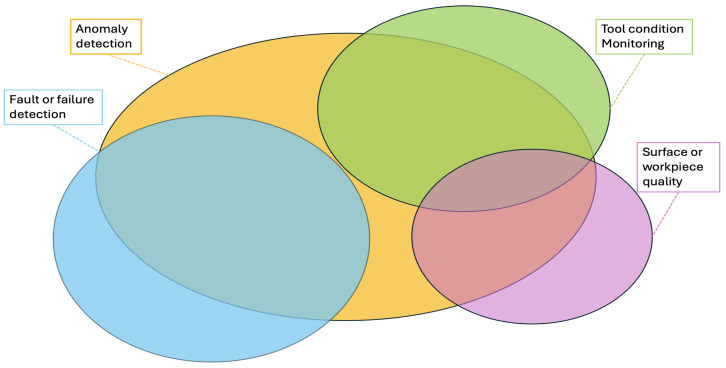
Predictive maintenance problems in Industry 5.0.

The analysis of the selected papers suggests that there is some variability in how problems are classified across different studies. In particular, certain distinctions between problem types are not always explicitly stated, and terminology may sometimes overlap or be used in a broad sense. This variability can make it challenging to precisely identify individual contributions and, in turn, to trace the progression of the field. As illustrated in Figure 26, many studies refer to anomaly detection; however, the term is sometimes used in the context of fault detection, while in other cases, it relates to TCM or surface quality issues. Additionally, there appears to be a notable interplay between TCM and surface quality. Some authors consider TCM the primary problem, as tool wear or suboptimal machining parameters directly affect surface quality. However, other studies addressed the issue by predicting the final quality of the workpiece, modeling parameters such as surface roughness based on tool life and machining conditions. This ambiguity in terminology and problem definition hinders a clear understanding of the field’s evolution. Therefore, it is crucial to establish a rigorous and consistent classification framework to facilitate comparisons between studies and support the scientific community in tracking advancements in the discipline.

Furthermore, the bibliometric analysis conducted in this study has also provided answers to the additional research questions formulated in Section 2.5. Specifically, it has helped to elucidate.

RQ1: What are the most common sustainability-related keywords and concepts linked to predictive maintenance in machining?

As demonstrated by the WordCloud in Figure 15, the keyword analysis revealed relevant terms associated with the three dimensions of sustainability: economic, environmental, and social aspects. A strong focus on the economic and environmental dimensions emerged from the analyzed papers, including an extensive analysis of production and maintenance costs and energy usage. These economical and environmental benefits can be achieved by constructing predictive models based on non-contact sensors, such as microphones for the acquisition of sound signals. Non-contact process monitoring could be a powerful tool to prevent improper usage of cutting tools, reduce electrical energy consumption and consequently reduce production costs [86]. The use of tool wear predictive models has the potential to enhance the efficiency and economic viability of industrial environments. By determining the optimal moment for replacing the cutting tool, the environmental and economic impact can be optimized. If the tool is worn to a certain degree and continues to be used, the machining quality and efficiency obviously decrease, resulting in product waste [87]. Conversely, premature replacement of a cutting tool can lead to an increase in production costs and machine downtime, thereby affecting the efficiency of the process. On the other hand, predictive models can also be developed for the purpose of machine failure prediction. These models assist in the scheduling of a suitable maintenance policy, thereby reducing the necessity for urgent and stressful maintenance interventions, as well as the risk of accidents related to the sudden breakage of a cutting tool or a component of industrial machinery. This, in turn, improves the social dimension of Sustainability 5.0. Despite this salient social aspect, only a limited number of studies have focused on it to date, suggesting a significant gap to be addressed in future research.

RQ2: What are the most prevalent AI techniques used in predictive maintenance for machining processes?

As both the WordCloud in Figure 14 and keywords in the Motor theme “learning systems” in Figure 25 show, DL techniques were the most prevalent. They have demonstrated high prediction performance for industrial problems by analyzing audio or AE signals. In particular, convolutional neural networks emerged as the most widely used DL technique. However, the main advantages of neural networks are related to the fact that they can process incoming data automatically, without a pre-processing step, as opposed to sensor data. Among the potential pre-processing tasks applicable to audio signals, their conversion into images emerged as the most predictive. This conversion can be achieved through the implementation of various techniques. The most used are the Fourier Transform and the Wavelet Transform. On the other hand, support vector machines were the most commonly used of the traditional ML approaches.

RQ3: Which specific manufacturing processes have been most frequently addressed in predictive maintenance studies?

The milling process was the most frequently analyzed. For all the industrial problems illustrated in Figure 26, several predictive maintenance models were tested in the literature. The milling process plays a crucial role in manufacturing [88]. It can accurately model the desired shapes and sizes of a machined workpiece, a capability that has been further enhanced with the advent of CNC machines. Subsequent processes include grinding and turning. Additionally, more studies investigated the failure of components of equipment to process these types of machining operations, as evidenced by the DCASE (Detection and Classification of Acoustic Scenes and Events) community’s establishment of an annual challenge concerning machine condition monitoring.

RQ4: How are IoT audio sensors integrated into predictive maintenance systems, and what challenges are associated with their application?

**Figure 27:**
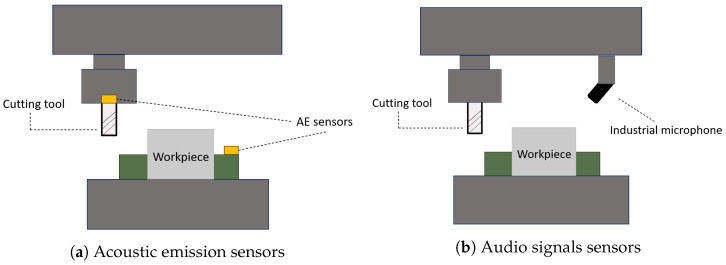
Possible positions of sensors in milling process.

In the scientific literature, when the subject of sound signals for industrial machines is discussed, two particular types of signal were identified: acoustic emissions, and audio signals. The primary distinction between the two lies in the type of sensor utilized for acquisition. In the case of acoustic emission, the sensor must be mounted internally within the cutting tool or the worktable, a configuration that can hinder the process if not properly aligned. Conversely, audio signals can be acquired with microphones positioned close to the work area, without necessitating any alterations to the machine’s configuration. An example of a sensor configuration for milling process is shown in Figure 27a for acoustic emissions and in Figure 27b for audio signals. However, acoustic emissions exhibit greater sensitivity to tool wear progression compared to audio signals, particularly in environments characterized by significant noise levels. In such cases, the preprocessing of audio signals can be more challenging than that of acoustic emissions.

In a real industrial environment, characterized by the presence of multiple machines operating simultaneously, high vibrations and temperatures, and significant external noise, aspects such as sensor applicability and system compatibility become crucial for the selection of the sensor to be adopted. Indeed, these factors can strongly influence the performance of AI algorithms trained on the signals acquired for the prediction of the failure or wear of materials. Acoustic emissions sensors are preferred in high-noise contexts. This is due to the fact that their required internal position can offer a degree of protection against noise. Conversely, identifying the correct position to avoid hindering the entire machining process may present a significant challenge. Meanwhile, microphones used for audio signal acquisition can be easily positioned near the working area. It should be noted, however, that attention to the orientation of the microphone should be exercised, as this can influence the performance of prediction models. Furthermore, the presence of external noise may necessitate the use of signal processing techniques, for noise filtering to ensure a high level of prediction performance. Finally, it is necessary to consider system compatibility, as this can increase the complexity of system integration and the associated costs. Acoustic emission sensors can require specific hardware components for their implementation. Conversely, microphones are generally more easily integrated, but their direct synchronization with the machining parameter configuration can be challenging.

RQ5: What is the geographical distribution of research efforts in predictive maintenance for machining processes?

Figure 17 shows that China is the leading country in publications concerning the construction of predictive maintenance systems based on the combination of IoT audio sensors and AI techniques. The number of citations obtained by China is close to double the number obtained by the USA. Figure 16 indicates that China published the highest number of papers in this field, followed by the USA, India, and Germany. Meanwhile, Italy is in fourth position in terms of the number of citations. Additionally, the analysis presented in Section 3.2.4 provides evidence of the existence of divergent trends in research focus across different countries.

RQ6: What publicly available datasets exist for the development of predictive maintenance systems, and how effectively do they support research and industrial applications?

As listed in Table 7, some datasets were identified as benchmark datasets from the analysis of scientific papers selected in this review work, with some of these datasets comprising a combination of others. However, the limited number of publicly available datasets highlights a critical issue in this domain. Companies, due to concerns related to privacy or competitiveness, are often reluctant to share their proprietary data. This presents a significant challenge for the field, as the lack of accessible datasets, particularly those collected from real industrial environments, obstructs the development of effective predictive models.

As previously emerged from the answer to RQ1, neural networks have consistently demonstrated the highest predictive performance for machining process problems through training them on simulated or laboratory datasets. However, as is well known, these techniques require large amounts of data for proper training. The scarcity of real-world industrial datasets thus limits the ability to develop and validate robust models that can generalize effectively to real manufacturing conditions. Addressing this challenge is crucial for advancing predictive maintenance systems and ensuring their successful integration into Industry 5.0 frameworks.

RQ7: Which variables are commonly monitored in predictive maintenance?

From an analysis of the top 10 most cited articles and available datasets, as representative of the best knowledge in the field, the target variables monitored and selected for the construction of predictive models were identified. The selected articles encompassed all maintenance problems for machining operations, as illustrated in Figure 26. Furthermore, heterogeneity was observed in the selection of the target variables, despite the consideration of a similar class of problem.

As was noted in the previous review work [27], the problem of TCM for milling process was formulated as both a regression and a classification problem in the relevant literature. The two available datasets, PHM2010 and NASA milling dataset, are related to the TCM problem in milling and both propose to solve a regression problem with the objective of predicting the flank wear and/or Remaining Useful Life (RUL) of the tool. While both datasets describe the same phenomenon of tool wear progression, they propose different target variables. However, these two target variables are strongly related to each other. A high value of flank tool wear can be translated as a low value of RUL. Consequently, solving one problem rather than the other would lead to the same goal, i.e., building a useful tool for the operator to identify the best time to replace the cutting tool. The difference between the two approaches proposed in the literature could be in the deployment phase in which the operator uses the automated tool, having to understand the response provided. Obtaining a flank tool wear value as a response, the operator would have to define the maximum wear value against which to replace the tool. Conversely, the RUL value could be defined as a percentage value, with a value close to 0 equivalent to a tool near the end of its useful life. In addition, some studies transformed these numerical target variables into a categorical, defining a range of values and solving a classification problem [53,59,62].

The same approaches were used in the literature for TCM of other machining operations. Pandiyan et al. [34] solved the problem of tool condition monitoring for a grinding process by categorizing the target variable and consequently solved a classification problem. Kuntoğlu and Sağlam [30] observed the flank tool wear of a turning cutting tool and solved the regression problem for its prediction.

In contrast, when the issue of surface quality was addressed, the majority of studies utilized numerical surface roughness measurement as the target variable, thereby resolving a regression problem [31].

Finally, concerning the failure prediction problem, both regression and classification approaches were considered in the literature, by defining a numerical target variable (i.e., RUL [32]) or a categorical target variable (i.e., Normal or Abnormal [39]).

A schematization of all the approaches used in the literature for the problems addressed is provided in Table 10.

Finally, a widespread usage of the sensor fusion methodology emerged from the selected papers [30,54,89,90,91,92,93,94,95,96,97]. The most used signals fused were vibrations, force, and acoustic emissions. Studies demonstrated that the usage of a single sensor can exhibit some limitations in detecting sensitive faults. A multi sensor approach can overcome this limitation by combining the advantages of all the sensors used. Contrarily, considering the sustainability goals of Industry 5.0, the use of such sensors can result in elevated costs for the implementation, disadvantaging their use in real industrial practice.

## 5. Conclusions

The present study undertook a bibliometric analysis in the field of predictive maintenance for machining operations, by considering the combination of IoT audio sensors and AI techniques as key elements for the construction of predictive models. A total of 304 papers were selected from Scopus and Web Of Science databases. The selected works belong in the context of Industry 5.0, in which the main goal is to improve all industrial outcomes related to the sustainability term, i.e., cost saving, energy efficiency, and waste reduction. The analysis yielded a high level of scientific interest. Audio sensors, a cost-effective type of sensor, exhibited high sensitivity to changes in industrial environments, suggesting their potential application in the construction of a predictive model for industrial problems. The findings of the study demonstrated the efficacy of prediction performance for tool condition monitoring, fault detection, and surface quality problems in all machining operations (milling, grinding, turning, etc.). A higher sensitivity to the two sustainability dimensions of economic and environmental aspects was exhibited in the literature, with considerable efforts being made to reduce costs and optimize energy consumption. However, only a limited number of studies have begun to address the third dimension of the social aspect, which relates to the well-being and security of operators. This reflects a significant gap in the literature for this field. In light of the emergent paradigm of Industry 5.0, it is imperative for the scientific community to make greater efforts to incorporate this social aspect into future research, thereby facilitating advanced studies. Human-centric approaches, prioritizing worker safety, well-being, and job satisfaction must be emphasized, in conjunction with economic and environmental goals. Achieving this objective necessitates interdisciplinary collaboration, utilizing technologies such as artificial intelligence, robotics, and smart systems to establish a more balanced and ethical industrial ecosystem, in accordance with Industry 5.0 principles. The leading country is China, followed by the USA. An absence of any significant inclination towards collaborative endeavors emerged from the analysis, both between different states and between companies, given the small number of publicly available datasets. A high number of publications were focused on the tool condition monitoring problem, especially for the milling process. Finally, utilization of the sensor fusion methodology is recommended to overcome the limitations of single sensors. However, it is important to note that this methodology is associated with a key disadvantage, which is the requirement for intrusive sensors, such as accelerometers or dynamometers. Conversely, the exclusive utilization of audio sensors can provide a cost-effective, non-intrusive solution for industrial machines, making them a desirable option in manufacturing environments.

Future research should concentrate on the use of this type of sensor and on conducting in-depth analyses of their use, to identify the best configuration to adopt for each type of industrial problem in each machining operation. Understanding the full potential of these sensors requires a comprehensive investigation, taking into account all factors that characterize the industrial context. These factors can significantly influence the performance efficiency of sensor systems defined for the predictive maintenance of machining processes. In parallel, future research should explore the integration of these sensor systems with digital twin technologies, enabling real-time monitoring, simulation, and analysis of the machining processes and creating more accurate and dynamic predictive models for industrial machines. Sensor data can continuously inform and update digital twin models, allowing continuous optimization of the machining processes and, consequently, the reductions in waste, energy consumption, downtime, and production costs.

## Figures and Tables

**Table 1:** Maintenance-related impacts on sustainability dimensions.

Economic Dimension	Environmental Dimension	Social Dimension
Reduction of costs	Reduction of hazardous emissions	Safe and Healthy working conditions
Prediction of unplanned downtime	Reduction of produced waste	Prediction of accidents
Reduction of waste	Efficient energy usage	Prediction of incidents
Increase in plant productivity	Effective resource consumption	Training for right maintenance actions
Increase in useful life of machinery	No wastage of stored materials	Reduction of risk factors
Maintenance time and costs reduction	Adaptive maintenance schedule	Easy communications

**Table 2:** Identified keywords for finding papers in databases.

Context	Intervention	Mechanism	Outcome
Where?	What?	How?	To get what?
In which context is the intervention embedded?	What is the main topic?	What is the medium?	What is the desired information?
**Machining** **operations:**	**Predictive** **maintenance** **systems:**	**IoT Audio** **sensors + AI:**	**Sustainability:**
Machining Milling Turning Drilling Threading Sawing Grinding Tool Wear Cutting Tool CNC Industrial Machines	Predictive Maintenance Condition-Based Maintenance Monitoring Fault Prediction Failure Prediction Anomaly Detection Tool Condition	Acoustic Emissions Sound Signal Audio Signal Microphone Machine Learning Artificial Intelligence Deep Learning Neural Network Data-driven LSTM CNN RNN Prediction	Sustainability Green Circular Economy Efficiency Waste Emissions Decarbonization Triple Bottom Line Carbon Footprint

**Table 3:** List of inclusion and exclusion criteria.

Inclusion Criteria	
1- Paper published in the period from 2014 to 2024.	
2- Paper from the fields of	
$\left[Scopus\right]$	$\left[Web\;of\;Science\right]$
Engineering; Computer Science; Mathematics; Material Science; Decision Sciences.	Engineering; Computer Science; Material Science; Automation Control Systems; Instruments Instrumentation; Operations Research Management Science; Metallurgy Metallurgical Engineering; Science technology other topics; Acoustics; Remote sensing; Mechanics.
**Exclusion criteria**	
1- Not written in English;	
2- Document types of Book, Book chapter, Editorial and Conference Review.	

**Table 4:** Research questions with their justification.

Research Question	Justification
What are the most common sustainability-related keywords and concepts linked to predictive maintenance in machining?	Identifying the most common sustainability-related keywords and concepts helps reveal the specific environmental, economic, and social aspects researchers prioritize, such as energy efficiency, waste reduction, circular economy practices, and operators well-being.
What are the most prevalent AI techniques used in predictive maintenance for machining processes?	Identifying the commonly used AI techniques will help in understanding the current technological focus and the most effective methods for achieving reliable and accurate predictions.
Which specific manufacturing processes have been most frequently addressed in predictive maintenance studies?	This question aims to determine whether the research has concentrated on particular processes (e.g., milling, turning, or grinding) and whether less-studied processes could benefit from further investigation.
How are IoT audio sensors integrated into predictive maintenance systems, and what challenges are associated with their application?	Understanding the integration of IoT audio sensors will shed light on their role in detecting anomalies and failures.
What is the geographical distribution of research efforts in predictive maintenance for machining processes?	Analyzing the geographical spread of studies can provide insights into regional research priorities and potential disparities in technological adoption or focus.
What publicly available datasets exist for the development of predictive maintenance systems, and how effectively do they support research and industrial applications?	Exploring available datasets can highlight their practical applicability, limitations, and opportunities for enhancing collaboration between academia and industry.
Which variables are commonly monitored in predictive maintenance?	Understanding the observed variables and modeling approaches helps clarify whether research primarily adopts classification or regression methods, offering insights into prevailing strategies and potential gaps.

**Table 5:** Core zone from the application of Bradford’s law.

Publication Type	Rank	Source
Journals	1	INTERNATIONAL JOURNAL OF ADVANCED MANUFACTURING TECHNOLOGY
	2	SENSORS
	3	IEEE ACCESS
	4	JOURNAL OF MANUFACTURING PROCESSES
	5	APPLIED SCIENCES (SWITZERLAND)
	6	MECHANICAL SYSTEMS AND SIGNAL PROCESSING
Conference proceedings	1	PROCEDIA CIRP
	2	PROCEDIA MANUFACTURING
	3	PROCEEDINGS OF SPIE—THE INTERNATIONAL SOCIETY FOR OPTICAL ENGINEERING
	4	MATERIALS TODAY: PROCEEDINGS
	5	ASME INTERNATIONAL MECHANICAL ENGINEERING CONGRESS AND EXPOSITION; PROCEEDINGS (IMECE)

**Table 6:** Top 10 most cited articles.

Article Title	Publication Year	Reference	TC * per Year
Investigation of signal behaviors for sensor fusion with tool condition monitoring system in turning	2021	[30]	26.60
Prediction of surface roughness based on a hybrid feature selection method and long short-term memory network in grinding	2021	[31]	24.00
Prognosis of Bearing Acoustic Emission Signals Using Supervised Machine Learning	2018	[32]	23.63
Automated bearing fault diagnosis scheme using 2D representation of wavelet packet transform and deep convolutional neural network	2019	[33]	21.43
In-process tool condition monitoring in compliant abrasive belt grinding process using support vector machine and genetic algorithm	2018	[34]	19.63
Tool wear monitoring in micromilling using Support Vector Machine with vibration and sound sensors	2021	[35]	19.20
Data-driven smart manufacturing: Tool wear monitoring with audio signals and machine learning	2019	[36]	19.00
Single-Sensor Acoustic Emission Source Localization in Plate-Like Structures Using Deep Learning	2018	[37]	15.88
A Hybrid Approach to Cutting Tool Remaining Useful Life Prediction Based on the Wiener Process	2018	[38]	12.25
Application of audible sound signals for tool wear monitoring using machine learning techniques in end milling	2018	[39]	11.38

* Total citations.

**Table 7:** Clusters from the thematic map.

Theme	Type	Meaning
Cutting tools	Motor theme	This is the largest and most detailed cluster, suggesting an emphasis on tool health diagnostics for several machining processes using different predictive techniques and a combination of signals
Learning systems	Motor theme	Highlights the role of AI techniques in monitoring and improving machining efficiency
Acoustic-emission	Motor theme	This cluster indicates the use of acoustic emissions as a primary method for monitoring tool wear and machine health
System	Basic theme	Reflects the structural and operational components of machining systems and how they are monitored using various signals and models
Neural-networks	Emerging theme	This cluster suggests a wide usage of deep learning techniques in the literature to solve problems related to predictive maintenance of industrial machines
Vibration signal	Basic theme	Suggests extensive use of vibration signals in addition to acoustic emissions
Prediction	Basic theme	Reflects the importance of the use statistical and ML methods to construct a predictive model for tool wear or machine failure
Force	Declining theme	Indicates the importance of force measurements for monitoring a machining process
Classification	Declining theme	Refers to the development of a predictive model using traditional ML and feature extraction techniques
Mechanisms	Niche theme	Highlights physical and mechanical principles governing tool wear and machine failure
Transform	Niche theme	Indicates the use of signal transformation techniques such as Fourier or wavelet transform
Fuzzy inference	Niche theme	Refers to fuzzy-logic-based approaches for decision-making in machining processes

**Table 8:** List of public datasets.

Dataset	Description	References	Papers
MIMII Dataset	A sound dataset of industrial malfunctions useful for constructing methods for fault detection. It contains sound generated by valves, pumps, fans, and side rails.	[40]	[41,42,43,44,45,46,47,48,49,50,51]
PHM 2010	A conference data challenge from the PHM society focused on RUL estimation for high-speed CNC milling machine cutters using dynamometer, accelerometer, and acoustic emission data.	[52]	[53,54,55,56,57,58,59,60,61,62]
NASA milling wear	Contains registrations of the wear progression for a milling process for different speeds, feeds, and depth of cut, monitored by three different types of sensors (acoustic emission sensor, vibration sensor, current sensor).	[63]	[53,62,64,65,66]
DCASE 2020 task 2	An anomalous sound detection dataset consisting of a mix of normal ToYAdamos and MIMII sound signals.	[67]	[44]
DCASE 2022 task 2	The follow-up to the DCASE 2020 task 2, with a focus on domain generalization	[68]	[69,70]
DCASE 2023 task 2	The follow-up to the DCASE 2022 task 2, with a focus on the first-shot problem	[71]	[69]

**Table 9:** List of literature reviews.

Reference	Year	Area	Keywords	Time Frame
[76]	2024	TCM	- tool condition monitoring; - industrial IoT; - machine learning; - industry 4.0; - CNC turning process; - signal processing	1950s–2024
[80]	2024	self-powered sensor systems	- self-powered sensors; - piezoelectricity; - triboelectricity; - thermoelectricity; - hybrid nanogenerator	most relevant research up to 2024
[81]	2023	Grinding wheel condition monitoring	- artificial neural network; - condition monitoring; - convolutional neural networks; - grinding wheel	most relevant research up to 2023
[79]	2022	Noise in High-Speed Cutting Machining	- cutting noise; - sound source analysis; - numerical recognition; - noise control; - condition monitoring	most relevant research up to 2022
[77]	2022	TCM in milling	- Conventional and artificial intelligence; - Direct and indirect monitoring Machining; - Measurement sensor systems; - Milling; - Tool condition monitoring (TCM)	most relevant research up to 2022
[78]	2023	Multi-fault diagnosis in rotating machines	- Industry 4.0; - Predictive maintenance; - Multi-fault diagnosis; - Multi-sensor data fusion; - Data acquisition; - Artificial intelligence; - Industrial rotating machines; - PRISMA guidelines	2011–2021
[82]	2022	TCM	- Artificial Intelligence; - Machining; - Tool condition monitoring; - Sensor; - Tool life; - Wear	2011–2021
[83]	2022	Chatter detection	- Chatter detection; - Signal processing; - Feature selection; - Classification; - Machining process	most relevant research up to 2021

**Table 10:** Schematization of resolution approaches by problem type.

Problem Type	Target Variable	Resolution Approach	
		Regression	Classification
Tool condition monitoring	Flank tool wear	✓	
	RUL	✓	
	Tool wear condition		✓
Surface quality	Surface roughness	✓	
Failure prediction	RUL	✓	
	Normal/Abnormal		✓

## Data Availability

All data presented in this manuscript are available on Scopus and Web of Science databases using the search query listed in the Section 2.
